# Concave-Octahedral Fe^2+^-Rich Fe-MOF/FU Nano-Blocks with Enhanced pH-Responsive Nanozyme Activity Toward Stimuli-Responsive Gels for Chemo-Chemodynamic Synergistic Therapy

**DOI:** 10.3390/gels11090750

**Published:** 2025-09-17

**Authors:** Desheng Wang, Changjin Xu, Laibing Wang, Herima Qi, Riqing Cheng, Liang Bao, Huiqing Guo, Shikui Wu

**Affiliations:** 1 College of Pharmacy, Inner Mongolia Medical University, Hohhot 010110, China; 2 Medical Innovation Center for Nationalities, Inner Mongolia Medical University, Hohhot 010110, China

**Keywords:** drug delivery, MOF, nanozyme activity, tumor microenvironment, multimodal therapy

## Abstract

Hydroxyl radicals (·OH) offer exceptional potential for cancer treatment through reactive oxygen species (ROS) amplification and apoptotic induction. However, conventional Fe-based metal–organic framework (Fe-MOF) nanomaterials are limited by inadequate Fe^2+^ concentrations, resulting in suboptimal Fenton catalytic performance. This study presents concave octahedral Fe-MOF nanomaterials with integrated bimetallic Fe/Zn centers through controlled solvothermal synthesis. The nanoplatform exhibits high specific surface area (559 m^2^/g) and 5-fluorouracil (5-FU) loading efficiency (58.7%). These structural properties establish it as a potential nanobuilding block for constructing stimuli-responsive gels. With optimized Fe^2+^ content (57.3%), the Fe-MOF material shows enhanced nanozyme-like activity (V_max_ = 4.58 × 10^−7^ M/s, K_cat_ = 1.83 × 10^−3^ s^−1^) for H_2_O_2_-mediated ·OH generation. The Fe-MOF@FU demonstrates pH-responsive drug release (76.5% at pH 5.0) and glutathione (GSH) depletion, synergistically enhancing oxidative stress. Biocompatibility studies confirm safety, while in vitro investigations show remarkable anticancer activity against 4T1 cells with 17.8% viability, supporting its dual role as an independent therapeutic agent and a functional component for future gel-based delivery systems.

## 1. Introduction

The complexity and heterogeneity of the tumor microenvironment (TME) present significant challenges to cancer therapy [1,2,3]. The TME is typically characterized by low pH, elevated glutathione concentration, and high hydrogen peroxide (H_2_O_2_) expression [4,5,6]. These characteristics not only provide a conducive environment for tumor proliferation and metastasis but also offer opportunities for highly efficient and selective cancer therapy [3,7]. In recent years, therapeutic strategies based on oxidative-antioxidative imbalance have become a prominent research focus. Among these, chemodynamic therapy (CDT) utilizes Fenton and Fenton-like reactions to generate highly toxic hydroxyl radicals within tumor sites, thereby inducing oxidative stress damage and programmed cell death in tumor cells [8,9]. However, most current CDT systems suffer from limited catalytic efficiency, restricted rates of ·OH generation, and suboptimal biocompatibility, which together hinder their clinical translation [10,11,12,13]. Therefore, the development of nanomaterials with high catalytic efficiency, pH-responsive properties, and favorable biosafety represents an important trend in antitumor research [4,14,15,16].

Metal–organic frameworks (MOFs) have shown great potential in drug delivery and catalytic therapy owing to their high specific surface area, tunable porosity, and multifunctional properties [17,18,19]. Beyond standalone nanoparticles, MOFs serve as ideal nanobuilding blocks for constructing stimuli-responsive hydrogels, where their ordered pores and surface functionality enable precise control over drug loading and microenvironment-triggered release. The significance of pH-responsive materials lies in their ability to selectively activate drug release and catalytic behavior within the acidic tumor microenvironment (pH~6.5–7.0), while remaining stable under physiological conditions (pH 7.4), thus enhancing therapeutic specificity and reducing off-target effects. In particular, iron-based MOFs have emerged as ideal candidates for CDT due to their ability to catalyze H_2_O_2_ via the Fenton reaction, facilitating effective ·OH production [20,21]. Nevertheless, conventional Fe-MOFs often contain a limited proportion of Fe^2+^ species, resulting in suboptimal Fenton catalytic performance and an insufficient ·OH yield to disrupt the cellular redox balance [1,4,17,22,23]. Additionally, the high intracellular GSH concentration in tumor cells acts as a reducing agent, further scavenging ·OH and diminishing the therapeutic efficacy of CDT [24,25]. To overcome these limitations, it is crucial to develop Fe-MOFs enriched in Fe^2+^ species, possessing pH-responsive characteristics and capable of effectively modulating GSH levels. Such advanced nano-blocks would not only enhance standalone CDT efficacy but also provide critical functional components for next-generation therapeutic gels requiring integrated catalytic activity and smart drug release.

**Scheme 1:**
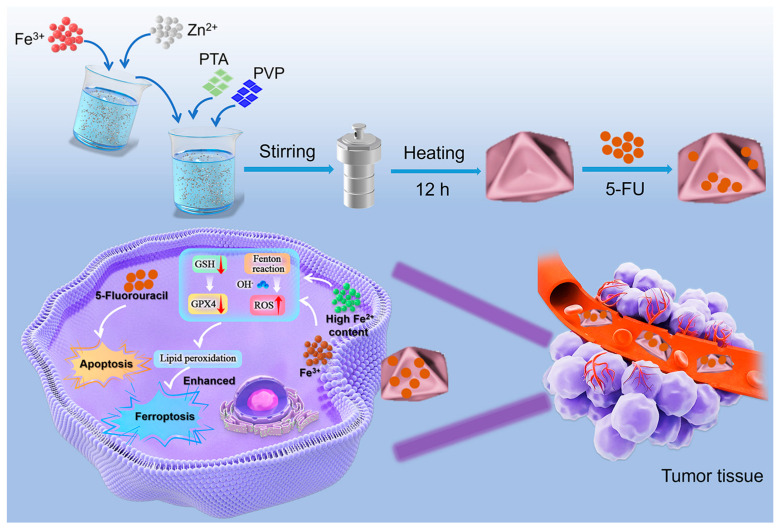
Schematic illustration of Fe-MOF@FU nanomaterial synthesis and the combined chemotherapy-CDT for tumor inhibition.

In this study, we successfully synthesized Fe-MOF nano-blocks with a concave octahedral morphology via a solvothermal method using Fe/Zn as bimetallic centers, as shown in Scheme 1. The term “concave octahedron” refers to a structure where the center of each face is inwardly curved, forming a complex three-dimensional architecture with higher surface area compared to conventional convex octahedra [26]. Polyvinylpyrrolidone (PVP) was introduced as a crystal growth modifier to precisely control the kinetic pathways, thereby enabling the formation of well-defined concave octahedral nanostructures. These structurally optimized particles serve as foundational units for future stimuli-responsive gel platforms. Their design features a high specific surface area (559 m^2^/g) and uniform mesopores to maximize drug and gel matrix integration. Additionally, an engineered Fe^2+^-rich composition (57.3%) provides potent catalytic functionality within gel networks. The particles also exhibit dual responsiveness to pH and GSH, enabling synergistic gel-MOF triggered therapy. The resulting material exhibits significantly enhanced drug loading efficiency (5-FU: 58.7%) and remarkable nanozyme activity (V_max_ = 4.58 × 10^−7^ M/s, K_cat_ = 1.83 × 10^−3^ s^−1^). Furthermore, the Fe-MOF@FU composite demonstrates programmable pH-responsive drug release (76.5% at pH 5.0) along with GSH depletion capability, while biocompatibility assessments confirm its clinical potential. Therefore, the synthesized Fe-MOF inorganic nanomaterial functions as a versatile support with tailored structural and catalytic properties, providing a solid foundation for constructing advanced gel-based delivery systems in future applications.

## 2. Results and Discussion

### 2.1. Characterization of the As-Prepared Fe-MOF Materials

**Figure 1:**
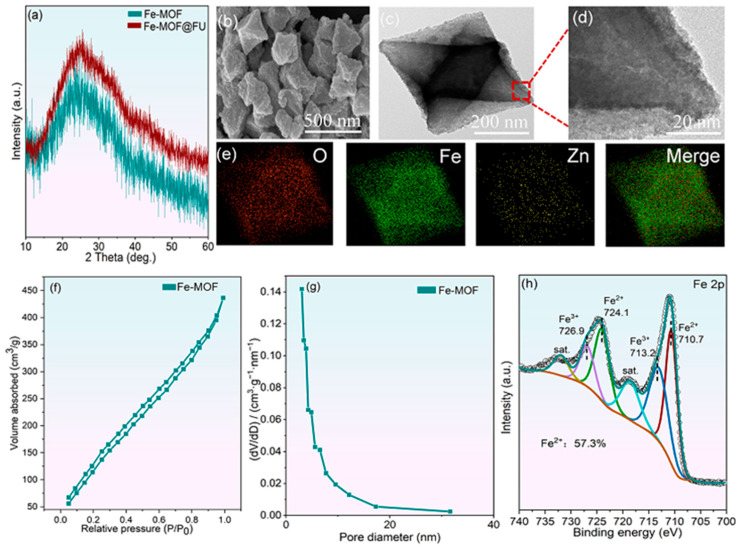
Structural characterization of the Fe-MOF and Fe-MOF@FU. (**a**) XRD pattern. (**b**) SEM image. (**c**,**d**) TEM images. (**e**) HAADF-STEM image and corresponding elemental mapping (Fe, O, Zn). (**f**) N_2_ adsorption–desorption isotherm. (**g**) Fe-MOF pore size distribution. (**h**) Fe 2p XPS spectrum.

Fe-MOF nanoparticles with a hollow porous structure and concave octahedral morphology were successfully synthesized via a solvothermal method using Fe^3+^ and Zn^2+^ as the metal center and PTA as the organic ligand. The XRD pattern (Figure 1a) exhibits characteristic diffraction peaks within the 5–20° range, consistent with literature reports, confirming the successful synthesis of the target Fe-MOF structure [27,28]. After loading with 5-FU, the Fe-MOF@FU composite shows nearly identical peak positions and FWHM values without any additional crystalline peaks corresponding to 5-FU, indicating that the drug was highly dispersed within the MOF matrix and that the original framework remained intact. Notably, the observed peak broadening indicates low crystallinity of the material. This structural feature is crucial as it suggests the framework is more prone to dissociation under physiological conditions, particularly when influenced by the TME [18,19]. This promoted degradation is expected to accelerate the metabolic clearance of the material components, thereby significantly enhancing the biocompatibility of Fe-MOF [29].

SEM characterization (Figure 1b) reveals that the Fe-MOF nanoparticles display a regular concave octahedral morphology with an average size of approximately 500 nm. Furthermore, TEM analysis (Figure 1c,d) uncovered abundant mesoporous structures within the material, a porous characteristic anticipated to significantly enhance its drug loading capacity [20,30]. Additionally, HAADF-STEM elemental mapping (Figure 1e) confirms the uniform distribution of Fe, O and Zn elements throughout the particles. The composition is as follows: oxygen (O) accounts for 34%, iron (Fe) for 63%, and zinc (Zn) for 3%. Furthermore, DLS measurements conducted over 5 days in two different media show minimal change in particle size, confirming the high stability of the material (Figure S1).

To evaluate the specific surface area and pore structure characteristics of Fe-MOF, N_2_ adsorption–desorption tests were conducted. The resulting isotherm exhibits typical Type IV characteristics with an H3-type hysteresis loop in the relative pressure (P/P_0_) range of 0.3–1.0 (Figure 1f), conforming to the IUPAC definition for slit-shaped mesoporous materials. Brunauer–Emmett–Teller (BET) specific surface area analysis determined the surface area of Fe-MOF to be 559 m^2^/g. This value is comparatively higher than those reported in many previous studies, which can be attributed to the unique concave octahedral structure of the material, providing a larger accessible surface area (Table S1). Furthermore, pore size distribution analysis indicates that the pore diameters were primarily concentrated within the range of 3–20 nm (Figure 1g). Critically, this well-defined meso-porosity and ultrahigh surface area establish an ideal architectural foundation for integrating Fe-MOF as functional nano-blocks within hydrogel matrices. This high specific surface area combined with the abundant mesoporous structure not only provides ample sites for drug loading but also creates favorable channels for the effective diffusion of drug molecules, suggesting the potential application value of this material in the field of drug delivery [27].

The chemical state of the Fe element in Fe-MOF was analyzed using XPS (Figure 1h). The Fe 2p spectrum, after peak fitting, comprised six sub-peaks. The high-resolution Fe 2p XPS spectrum could be deconvoluted into two characteristic peaks at binding energies of 710.7 eV and 713.2 eV, corresponding to Fe^2+^ and Fe^3+^ species, respectively. The relative content of Fe^2+^ was quantitatively determined based on the area ratio of these peaks using the formula: Fe^2+^(%) = [A(Fe^2+^)/(A(Fe^2+^) + A(Fe^3+^)] × 100%. The calculated Fe^2+^ content reached 57.3%, which is significantly higher than those reported in similar Fe-MOF-based catalysts in the literature [28,31,32]. This high concentration of Fe^2+^ species is essential for efficiently catalyzing the Fenton reaction with H_2_O_2_, thereby enhancing the yield of ·OH radicals. This increased radical production strengthens the cytotoxic effect on tumor cells, inducing their apoptosis.

### 2.2. In Vitro Catalytic Activity and Drug Loading/Release Behavior

**Figure 2:**
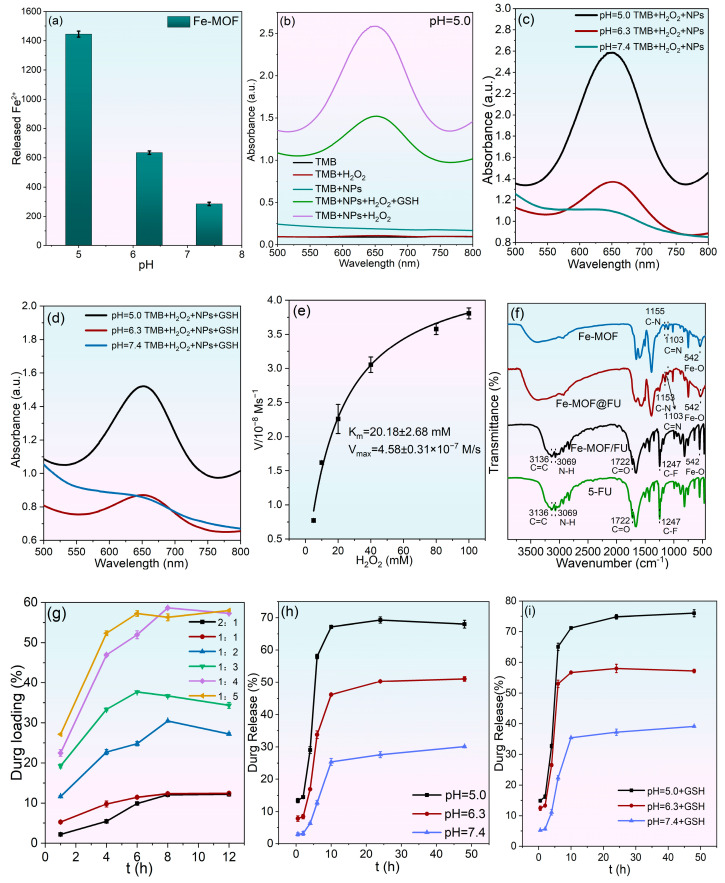
(**a**) The release amount of Fe^2+^ from Fe-MOF materials under different pH conditions. (**b**) UV–vis absorption spectra of TMB after reaction with Blank, H_2_O_2_, NPs, NPs + H_2_O_2_ + GSH, and NPs + H_2_O_2_ (where NPs represent Fe-MOF materials). (**c**) UV–vis absorption spectra of TMB after reaction with NPs + H_2_O_2_ under different pH conditions. (**d**) UV–vis absorption spectra of TMB after reaction with NPs + H_2_O_2_ + GSH under different pH conditions. (**e**) Michaelis–Menten kinetic analysis. (**f**) FTIR of 5-FU, Fe-MOF, Fe-MOF@Fu and Fe-MOF/FU. (**g**) Drug loading performance of Fe-MOF materials with different mass ratios (m_material_:m_5-FU_) over time. (**h**) 5-FU release profiles of Fe-MOF@FU materials in PBS solution at different pH values. (**i**) 5-FU release profiles of Fe-MOF@FU materials in PBS and GSH mixed solutions at different pH values.

The Fenton reaction, which converts endogenous H_2_O_2_ into highly toxic ·OH catalyzed by Fe^2+^, is an effective strategy for elevating intracellular ROS levels and inducing apoptosis. Rapid release of Fe^2+^ is crucial for achieving efficient burst generation of ·OH, thereby promptly inducing tumor cell apoptosis [33,34,35]. Therefore, to assess the stimuli-responsive release properties of the synthesized Fe-MOF material, we measured its Fe^2+^ release under simulated physiological conditions (pH 7.4) and the TME. As shown in Figure 2a, Fe-MOF exhibited only minimal Fe^2+^ release under neutral conditions (pH 7.4), while significantly enhanced Fe^2+^ release was achieved under acidic conditions. This result confirms the material’s ability to specifically respond to the acidic stimulus of the TME for on-demand controlled release of Fe^2+^.

To evaluate the capacity of Fe-MOF to catalyze the decomposition of H_2_O_2_ and generate ·OH across a physiologically relevant pH gradient—from the acidic lysosome (pH~5.0) [36] through the tumor microenvironment (pH~6.5–7.0) to normal tissue (pH~7.4) [17] a foundational chromogenic assay using TMB was performed (Figure 2b). In the control groups (TMB alone, TMB + Fe-MOF, and TMB + H_2_O_2_), no significant color change was observed, confirming that neither Fe-MOF nor H_2_O_2_ alone induces oxidation under acidic conditions. In contrast, the mixture of TMB + Fe-MOF + H_2_O_2_ immediately produced a distinct blue color, directly demonstrating the efficient catalytic generation of ·OH by Fe-MOF at pH 5.0. Given that high intracellular GSH levels in tumor cells can compromise oxidative therapeutic efficacy, the effect of GSH was further investigated at the same pH 5.0 (Figure 2b): although the ·OH signal (blue intensity) weakens after GSH addition, the system could still effectively consume GSH and maintain a certain level of ·OH production, indicating its potential for enhancing CDT by depleting GSH [37,38,39,40].

Building on the established basic catalytic behavior and GSH scavenging effect at pH 5.0, the influence of different pH values was further explored to systematically investigate ·OH generation efficiency and the response mechanism to reductive microenvironments (Figure 2c,d). The three key pH values (7.4, 6.3, and 5.0) were strategically selected to accurately mimic the core physiological microenvironments along the entire nanocarrier delivery pathway: from circulation in normal tissue (stability, pH 7.4) → accumulation and enrichment in the tumor microenvironment (TME, targeting trigger, pH~6.5) [17] → cellular internalization (uptake) → to endo-lysosomal release (functional fulfillment, pH 5.0) [36]. This systematic investigation is crucial as the designed pH-sensitive material is engineered to sequentially respond to the mildly acidic TME (pH~6.5–7.0) for enhanced targeting, and to the more acidic endo-lysosomal compartments (pH~4.5–5.0) for rapid and thorough drug release. Results show that under pH 5.0, simulating the TME, the TMB + Fe-MOF + H_2_O_2_ system exhibit the strongest ·OH generation capacity (highest absorbance); notably, even in the TMB + Fe-MOF + H_2_O_2_ + GSH system containing GSH, a detectable level of ·OH signal (absorbance) was still observed at pH 5.0. Although its intensity was lower than that of the GSH-free system, this result is fully consistent with the partial GSH scavenging effect observed in Figure 2b at the same pH, further confirming the system’s ability to resist GSH consumption and sustain ·OH generation under acidic conditions.

Given Fe-MOF’s capability to rapidly catalyze H_2_O_2_ to produce ·OH in the tumor microenvironment, its kinetic behavior conforms to the Michaelis-Menten equation, demonstrating significant POD-like activity. Therefore, its Michaelis constant (K_m_) and maximum reaction rate (V_max_) were determined, with results shown in Figure 2e. Compared to literature values [15,41,42,43,44,45], the Fe-MOF material exhibits a higher K_m_ value (20.18 ± 2.68 mM), a higher V_max_ value (4.58 × 10^−7^ M/s) and a higher catalytic efficiency (K_cat_ = 1.83 × 10^−3^ s^−1^) for ·OH generation, further confirming its potential as a highly efficient nanozyme under acidic conditions.

Leveraging Fe-MOF’s high specific surface area and abundant mesoporous structure, 5-FU was selected as a model drug to evaluate its drug loading capacity. Drug molecules primarily adsorb onto the material surface via weak interactions such as van der Waals forces and hydrogen bonding. To verify successful drug loading and investigate its state, Zeta potential and FT-IR analyses were performed. Zeta potential testing (Figure S2) reveals that the potential of Fe-MOF was 19.8 ± 1.74 mV, which significantly increases to 23.2 ± 2.12 mV after drug loading (Fe-MOF@FU), indicating successful adsorption of 5-FU molecules onto the material surface. FTIR spectroscopy (Figure 2f) reveals three characteristic peaks of the Fe-MOF at 1155, 1103, and 542 cm^−1^, which are attributed to its framework vibrations. In contrast, pure 5-FU exhibits a distinct absorption peak at 1247 cm^−1^, corresponding to the C–F stretching vibration [17,46]. The spectrum of Fe-MOF@FU shows no significant changes compared to Fe-MOF. No characteristic vibration peaks of 5-FU were detected. In contrast, the physical mixture Fe-MOF/FU clearly displays the characteristic peaks of 5-FU. These results indicate that 5-FU is primarily loaded in a highly dispersed state on the material’s pore surfaces, rather than forming crystals or aggregates.

To optimize the drug loading performance of Fe-MOF, the influence of the feeding ratio on its drug loading efficiency was systematically investigated (Figure 2g). Experimental results show that the drug loading efficiency increased with the increase in the 5-FU feeding ratio. Based on the standard curve (Figure S4), at a feeding ratio of 1:4, Fe-MOF achieves a drug loading capacity of 58.7% within 8 h. This value is notably higher than those reported in many previous studies (Table S1), which can be attributed to the large specific surface area provided by the unique concave octahedral structure of the Fe-MOF, facilitating enhanced drug adsorption. This maximized drug payload is particularly valuable for future hydrogel composites, where Fe-MOF nano-blocks will serve as high-density drug reservoirs to overcome the limited loading capacity of conventional gel matrices. However, further increasing the feeding ratio or extending the loading time (>8 h) does not significantly improve the loading efficiency. Therefore, considering both loading performance and economic feasibility, a feeding ratio of 1:4 and a loading time of 8 h were selected as the optimal loading conditions.

Furthermore, based on the characteristics of the TME, the drug release behavior of Fe-MOF@FU was evaluated using PBS solutions simulating physiological conditions (pH 5.0–7.4) (Figure 2h). The material exhibits significant pH-responsive release characteristics. Within 48 h, the cumulative release rate of 5-FU reached 68.2 ± 1.19% under acidic conditions (pH 5.0), but only about 30% was released at neutral pH (7.4). Further study (Figure 2i) shows that when high concentrations of GSH (simulating the TME reductive environment) were added to the PBS, the cumulative release of 5-FU at pH 5.0 further increases to 76.5 ± 1.2%, still maintaining a high drug release rate. These results confirm that Fe-MOF@FU can synergistically respond to the low pH and high GSH concentration of the TME to achieve intelligent controlled drug release. This programmable release kinetics provides a critical design blueprint for developing tumor microenvironment-responsive hydrogel delivery systems [47].

### 2.3. Cellular Uptake and In Vitro Therapeutic Evaluation of Fe-MOF

**Figure 3:**
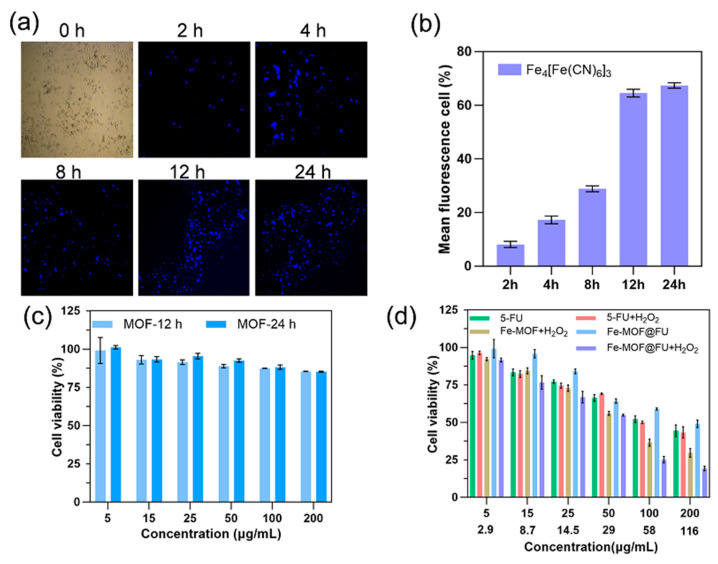
(**a**) Prussian blue staining images of 4T1 cells incubated with Fe-MOF for different durations (Scale bar: 20 μm). (**b**) Quantitative analysis of intracellular iron accumulation. (**c**) Viability of 4T1 cells treated with different concentrations of Fe-MOF for 12 h or 24 h. (**d**) Survival rate of 4T1 cells treated with 5-FU, 5-FU + H_2_O_2_, Fe-MOF + H_2_O_2_, Fe-MOF@FU, and Fe-MOF@FU + H_2_O_2_. Concentration axes: upper for Fe-MOF support and Fe-MOF@FU (5–200 μg/mL); lower for equivalent free 5-FU (2.9–116 μg/mL).

Cellular internalization is a prerequisite for the in vivo application of nanomaterials, and determining the optimal intracellular accumulation time is crucial for achieving maximum therapeutic efficacy [48]. This study detected intracellular iron ions in 4T1 cells by exploiting the characteristic reaction between K_4_[Fe(CN)_6_]) and Fe^2+^/Fe^3+^ to form a blue precipitate. As shown in Figure 3a, visible blue precipitate formed within the cells. The staining intensity increased progressively with longer incubation times and peaked at 12 h. Beyond this point, extending the incubation time did not significantly increase the intensity. Therefore, the optimal accumulation time of Fe-MOF in 4T1 cells was determined to be 12 h. Quantitative analysis results (Figure 3b) clearly demonstrate that the intracellular enrichment rate of the material reached its maximum after 12 h of incubation.

Biocompatibility is a core indicator for evaluating the clinical feasibility of nanotherapeutic materials. Cytotoxicity assays verified that Fe-MOF maintained excellent biocompatibility, as evidenced by cell viability remaining significantly above 80% even after co-incubation with 4T1 cells at a high concentration of 200 μg/mL for 12 and 24 h (Figure 3c). To validate the synergistic anti-tumor effect of Fe-MOF@FU within the TME, the survival rates of 4T1 cells under different treatment conditions were assessed using the CCK-8 assay (Figure 3d). The results reveal no significant difference in cell survival between the free 5-FU and the simulated TME group (H_2_O_2_ + 5-FU), indicating that H_2_O_2_ does not markedly alter drug sensitivity. In contrast, the CDT treatment group (Fe-MOF + H_2_O_2_) exhibits a significant decrease in cell survival, reaching approximately 28% at the highest concentration. This is primarily attributed to the highly abundant Fe^2+^ ions within the Fe-MOF material catalyzing the Fenton reaction to generate toxic ·OH radicals, thereby inducing cell apoptosis. Strikingly, when Fe-MOF@FU was combined with H_2_O_2_, cell survival dropped to its lowest level, around 17.8% at the highest concentration. This exceptional standalone efficacy positions Fe-MOF@FU nano-blocks as potent therapeutic components for future localized gel-based delivery systems. This pronounced cytotoxic effect stems from the synergistic action of ferroptosis and the chemotherapeutic efficacy of 5-FU. The drug-loaded system thus enhances tumor cell killing through dual mechanisms—simultaneously releasing the chemotherapeutic agent (5-FU) and catalytically generating ROS [49].

**Figure 4:**
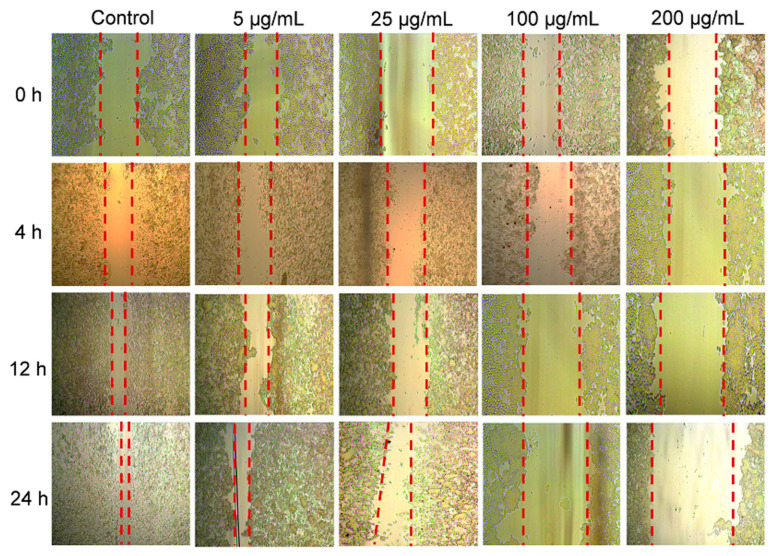
Wound healing in 4T1 cells after treatment with Fe-MOF@FU at varying concentrations and durations.

Furthermore, the impact of Fe-MOF@FU on the migratory capacity of 4T1 cells was evaluated using a wound healing assay. The results reveal that compared to the blank control group, the growth of 4T1 cells treated with Fe-MOF@FU was inhibited to varying degrees (Figure 4). At identical treatment time points, the inhibitory effect of Fe-MOF@FU on 4T1 cell migration exhibits a concentration-dependent manner, meaning the suppression of cell migration progressively intensified as the concentration of Fe-MOF@FU increased. These findings indicate that Fe-MOF@FU not only effectively kills tumor cells but also significantly suppresses their migratory ability, suggesting its potential value for anti-metastasis strategies. Such dual functionality is highly desirable for implantable hydrogel devices aimed at preventing post-surgical tumor recurrence.

**Figure 5:**
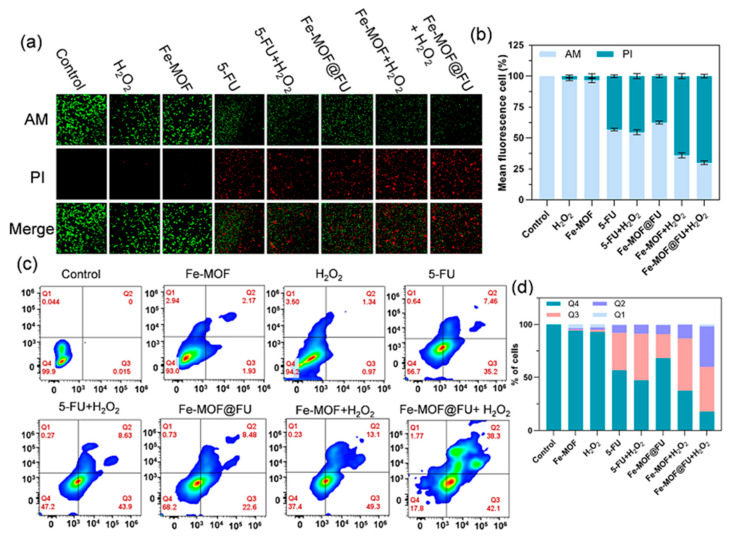
(**a**) Confocal laser scanning microscopy images of Calcein-AM/PI double-stained 4T1 cells under different experimental conditions (scale bar: 200 μm). (**b**) Semi-quantitative analysis of Calcein-AM/PI fluorescence intensity of 4T1 cells after different treatments. (**c**) Flow cytometry analysis of 4T1 cells stained with Annexin V-FITC/PI under different conditions. The quadrants Q1, Q2, Q3, and Q4 represent necrotic, late apoptotic, early apoptotic, and viable cells, respectively. The numbers in each quadrant indicate the percentage of corresponding cells. (**d**) Statistical histograms showing the percentages of cells in Q1, Q2, Q3, and Q4 quadrants after different treatments. Data are presented as mean ± standard deviation (mean ± SD, *n* = 3).

Building upon the CCK-8 cell viability results, the therapeutic effect was further visualized and validated using Calcein-AM/PI dual staining (Figure 5a). The results demonstrate that the control, H_2_O_2_, and Fe-MOF groups exhibit high-density green fluorescence (viable cells) with negligible PI red fluorescence (dead cells), indicating no significant membrane damage under basal conditions. In contrast, the 5-FU, 5-FU + H_2_O_2_, and Fe-MOF@FU groups show localized foci of PI red fluorescence, although viable cells remained predominant, confirming the incomplete cell killing achieved by chemotherapy alone. Strikingly, the CDT group (Fe-MOF + H_2_O_2_) and the combination therapy group (Fe-MOF@FU + H_2_O_2_) display extensive PI red fluorescence coverage with nearly absent green fluorescence. This observation signifies the complete disruption of cellular membrane integrity induced by Fe^2+^ ions from the material catalyzing the Fenton reaction, leading to massive apoptosis (consistent with the Fe valence states revealed by XPS). Quantitative analysis (Figure 5b) further reveals that the cell death rate in the combination therapy group increased by 1.7-fold compared to the optimal monotherapy group (5-FU) and by 1.1-fold compared to the CDT alone group, demonstrating that the chemo-CDT bimodal therapy maximizes the induction of tumor cell apoptosis through synergistic effects.

To quantitatively assess the ability of different treatment strategies to induce tumor cell apoptosis, classic Annexin V-FITC/PI flow cytometry was employed (Figure 5c,d). The results clearly indicate that cell viability remained above 90% in the blank control, H_2_O_2_, and Fe-MOF alone groups, with no statistically significant differences among them, confirming the good biocompatibility of the H_2_O_2_ concentration and Fe-MOF material used [22]. In contrast, conventional chemotherapy shows limitations. Specifically, the 5-FU alone, 5-FU + H_2_O_2_, and Fe-MOF@FU groups exhibit cell viabilities of 56.7%, 47.2%, and 68.2%, respectively, indicating moderate cytotoxicity. Notably, Fe-MOF-based CDT (Fe-MOF + H_2_O_2_) demonstrates potent pro-apoptotic capability, significantly reducing cell viability to 37.4%. This robustly proves Fe-MOF’s efficacy in catalyzing endogenous H_2_O_2_ within the tumor microenvironment to generate highly toxic ROS, enabling efficient cell killing. Crucially, the combination of chemotherapy and CDT, i.e., the combination therapy group (Fe-MOF@FU + H_2_O_2_), resulted in a sharp further decrease in cell viability to 17.8%. This value is markedly lower than all mono- or dual-therapy groups and represents a 19.6% reduction compared to the CDT group alone, fully revealing a powerful synergistic enhancement between the chemotherapeutic drug 5-FU and Fe-MOF-driven CDT. These results are highly consistent with the observations from the aforementioned Calcein-AM/PI live/dead staining assay, systematically confirming at the level of cellular apoptosis that the anti-tumor efficacy of the combined chemo-CDT strategy far exceeds the simple additive effects of any single modality. This provides compelling experimental support for developing highly effective anti-tumor nanomedicine platforms.

### 2.4. ROS Generation Capacity and Biocompatibility Assessment

**Figure 6:**
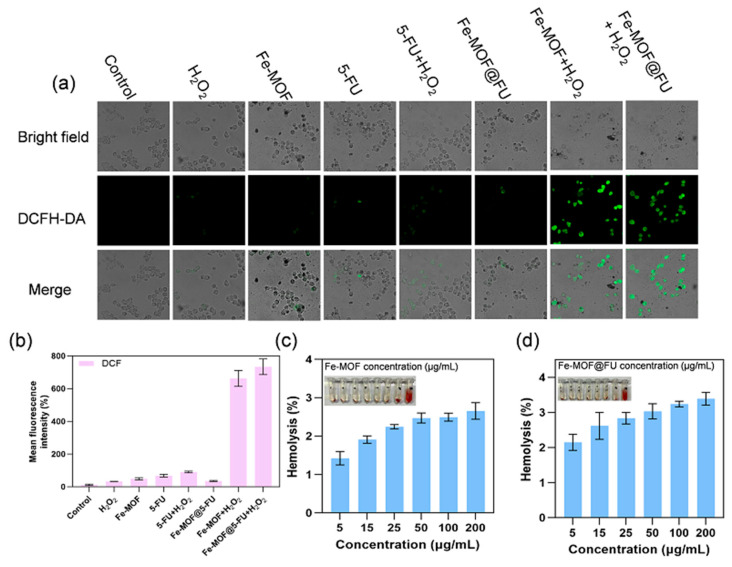
In vitro ROS generation capacity and hemocompatibility evaluation of different treatment groups. (**a**) Intracellular ROS levels in 4T1 cells of different groups detected by DCFH-DA fluorescent probe, with green fluorescence intensity indicating the relative ROS content. (**b**) Semi-quantitative analysis of intracellular ROS fluorescence intensity in each group. (**c**,**d**) In vitro hemolysis rates of different concentrations of Fe-MOF and Fe-MOF@FU materials, respectively.

Cellular redox homeostasis is a core regulatory mechanism maintaining metabolic balance [3]. Its disruption can compromise the functional integrity of critical biomacromolecules through pathways such as lipid peroxidation, protein carbonylation, and DNA oxidative damage, thereby triggering programmed cell death modes including ferroptosis, apoptosis, necroptosis, and pyroptosis. Building upon our previous finding of the Fenton-like catalytic activity of Fe-MOF nanomaterials towards H_2_O_2_ (generating highly cytotoxic · OH), this study employed the DCFH-DA fluorescent probe to quantitatively detect intracellular ROS levels. DCFH-DA can be specifically oxidized by ROS into the green fluorescent product 2′,7′-dichlorofluorescein (DCF), whose fluorescence intensity correlates positively with ROS concentration. As shown in Figure 6a,b, DCF fluorescence signals remains at low levels in the control group, H_2_O_2_ control group (exogenous H_2_O_2_ only), Fe-MOF alone group, 5-FU monotherapy group, and Fe-MOF@FU group. This indicates that only trace amounts of ROS are generated via non-enzymatic decomposition pathways under conditions lacking Fe^2+^ catalysis or sufficient H_2_O_2_ concentration. In stark contrast, a significant enhancement in DCF fluorescence intensity was observed in the CDT (Fe-MOF + H_2_O_2_) group simulating the tumor microenvironment (with exogenous H_2_O_2_ supply), confirming the efficient activation of the Fenton reaction and induction of a ROS burst by Fe-MOF. Further analysis reveals that the fluorescence intensity in the combination therapy group (chemo-CDT, Fe-MOF@FU + H_2_O_2_) was 1.1-fold higher than that in the CDT alone group. This enhancement may originate from the cellular stress effects induced by 5-FU chemotherapy. By interfering with nucleotide metabolism, 5-FU exacerbates acidification in the tumor microenvironment. This optimized Fenton reaction kinetics and enables cascaded ROS amplification, ultimately enhancing tumor cell killing.

To verify the biosafety of the materials, hemolysis assays were conducted (Figure 6c,d). The results demonstrate that after co-incubation with mouse red blood cell suspensions for 2 h across a concentration range of 5–200 μg/mL, the hemolysis rates of both Fe-MOF and Fe-MOF@FU remained consistently below 5%. This value is significantly lower than the safe hemolysis threshold (<5%) stipulated by the Chinese pharmacopoeia standard (Beijing China, 2023) for medical device biocompatibility. These data confirm that neither material induced significant hemolytic effects across the tested concentration range. This result supports their good hemocompatibility and indicates a solid biocompatibility foundation for potential intravenous administration. Therefore, this hemocompatibility is essential for intravenous administration of free nano-blocks or their potential leakage from biodegradable gel matrices.

**Figure 7:**
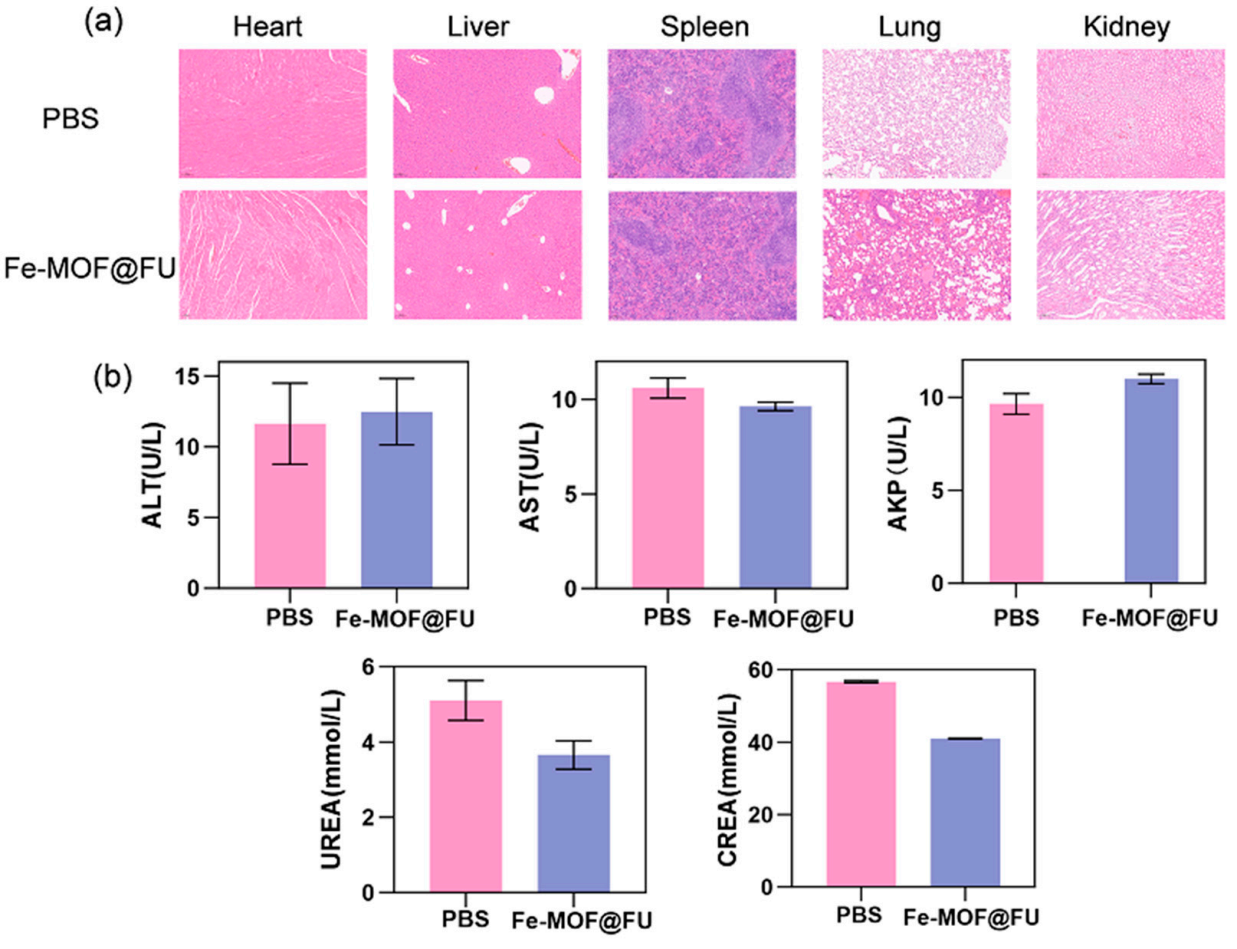
(**a**) Comparative H&E-stained sections of major organs from the PBS control group and the Fe-MOF@FU group 48 h post-treatment (Scale bar: 100 μm). (**b**) Analysis of serum biochemical markers: Liver function (Alanine aminotransferase, ALT; Aspartate aminotransferase, AST; Alkaline phosphatase, AKP) and kidney function (Urea, UREA; Creatinine, CREA) levels.

Histopathological evaluation of H&E-stained sections (Figure 7a) shows no significant differences in major organs—including the heart, liver, spleen, lung, and kidney—between the PBS control group and the group injected with 200 μg/mL Fe-MOF@FU. Key pathological features such as tissue architecture, cellular morphology, and inflammatory infiltration remain comparable across groups at 48 h post-injection. This indicates that Fe-MOF@FU at this therapeutic concentration does not induce observable organ toxicity or tissue damage. Further analysis of serum biochemical markers confirms that liver function indicators (ALT, AST, AKP) and kidney function indicators (UREA, CREA) in the Fe-MOF@FU group remained within normal physiological ranges (Figure 7b). Collectively, these results demonstrate that the Fe-MOF@FU nanomaterial exhibits excellent biocompatibility and minimal systemic toxicity risk.

## 3. Conclusions

In summary, we successfully developed a novel Fe-MOF nanoplatform with bimetallic Fe/Zn centers that overcome the limitations of traditional Fe-MOF materials in cancer therapy. The synthesized concave octahedral Fe-MOF exhibits superior physicochemical properties and remarkable 5-FU loading capacity. The rational incorporation of high-content Fe^2+^ (57.3%) endows the Fe-MOF material with superior Fenton catalytic efficiency (V_max_ = 4.58 × 10^−7^ M/s, K_cat_ = 1.83 × 10^−3^ s^−1^), enabling efficient ·OH generation for enhanced tumor therapy. The Fe-MOF@FU nanoplatform demonstrated pH-responsive drug release and effective GSH depletion, synergistically disrupting tumor redox homeostasis. In vitro studies confirmed excellent biocompatibility and remarkable anticancer efficacy against 4T1 breast cancer cells, reducing cell viability to 17.8% while inhibiting migration. The programmable TME-responsive functionality and high drug payload establish Fe-MOF@FU as both a promising nanoplatform for chemo-chemodynamic synergistic therapy and versatile nanoblocks for next-generation therapeutic hydrogels, offering a precise approach for cancer treatment.

## 4. Materials and Methods

### 4.1. Materials

Ferric chloride hexahydrate (FeCl_3_·6H_2_O), zinc nitrate hexahydrate (Zn(NO_3_)_2_·6H_2_O), *N*,*N*-dimethylformamide (DMF), and ethanol (CH_3_CH_2_OH) were purchased from Shanghai YiEn Chemical Technology Co., Ltd. (Shanghai, China). Potassium ferrocyanide (K_4_Fe(CN)_6_), terephthalic acid (PTA), and polyvinylpyrrolidone (PVP) were obtained from Shanghai Macklin Biochemical Technology Co., Ltd. (Shanghai, China). The average molecular weight of PVP is 58,000. 4T1 cells, RPMI 1640 medium, fetal bovine serum (FBS), trypsin, and penicillin-streptomycin solution were sourced from Thermo Fisher Scientific Inc. (Shanghai, China). Cell Counting Kit-8 (CCK-8), Annexin V-FITC/PI Apoptosis Detection Kit, calcein acetoxymethyl ester (Calcein-AM), propidium iodide (PI), and 2′,7′-dichlorodihydrofluorescein diacetate (DCFH-DA) were procured from Cyville Innovation Biotechnology Co., Ltd. (Wuhan, China). Dimethyl sulfoxide (DMSO) and 3,3′,5,5′-tetramethylbenzidine (TMB) were supplied by Beijing Dingguo Changsheng Biotechnology Co., Ltd. (Beijing, China). Phosphate-buffered saline (PBS), normal saline (0.9% NaCl solution), and 5-fluorouracil (5-FU) were purchased from Shanghai Yuanye Bio-Technology Co., Ltd. (Shanghai, China). All materials were applied in their original form with no additional purification procedures.

### 4.2. Synthesis of Concave Octahedral Fe-MOF

In this study, FeCl_3_·6H_2_O and Zn(NO_3_)_2_·6H_2_O serve as sources of Fe^3+^ and Zn^2+^ ions, which act as metal nodes within the MOF framework. PTA functions as the organic linker, coordinating with both Fe^3+^ and Zn^2+^ ions. The specific coordination of iron, particularly its location on tetrahedral framework positions, significantly influences the optical properties of the resulting MOF, often manifesting as distinct coloration [50]. Therefore, the role of solvents in facilitating this specific coordination environment requires careful consideration. PVP is employed to modulate crystal growth and control morphological development, while a mixed DMF/ethanol solvent system facilitates solvothermal synthesis and supports crystallization under synergistic conditions. Concave octahedral Fe-MOF was synthesized as follows: First, 46.0 mg of FeCl_3_·6H_2_O and 46.5 mg of Zn(NO_3_)_2_·6H_2_O were dissolved in 19.0 mL of a mixed solvent of DMF and CH_3_CH_2_OH under ultrasonication for 10 min until complete dissolution. Simultaneously, 9.6 mg of PTA and 387.0 mg of PVP were dissolved in 7.0 mL of a separate DMF/CH_3_CH_2_OH mixed solvent in a volume ratio of 5:3. The metal salt solution was then poured into the PTA-PVP mixture (mass ratio = 1:40) and stirred at room temperature for 2 h. The resulting mixture was transferred into a 100 mL Teflon-lined autoclave and heated at 120 °C for 12 h. After cooling naturally to room temperature, an orange precipitate was obtained, which was collected by centrifugation. This distinctive orange color indicates the specific coordination environment formed between Fe^2+^, Zn^2+^, and terephthalic acid within the mixed-metal MOF structure. The product was washed repeatedly with DMF and ethanol to remove impurities and finally vacuum-dried at 60 °C for 24 h to obtain porous concave octahedral Fe-MOF. In comparison to previous studies focused on single-metal configurations [49,51], the current system exhibits enhanced structural properties attributable to the cooperative effects of bimetallic centers and surfactant assistance.

### 4.3. Synthesis of Fe-MOF@FU Materials

The chemotherapeutic drug 5-FU was loaded into the porous concave octahedral Fe-MOF nanomaterial as follows: First, Fe-MOF was uniformly dispersed in a methanol-water mixed solvent to form a uniformly dispersed suspension. A quantified amount of 5-FU was added to this suspension, with the mass ratio of Fe-MOF to 5-FU systematically modulated (2:1, 1:1, 1:2, 1:3, 1:4, 1:5). The mixture was magnetically stirred at room temperature for gradient durations (1, 4, 6, 8, 12 h). After reaction completion, the drug-loaded precipitate was collected by centrifugation, and the supernatant containing residual drug was separated. The precipitate was washed three times with distilled water to thoroughly remove unadsorbed 5-FU. The final drug-loaded nanomaterial was designated Fe-MOF@5-FU (abbreviated as Fe-MOF@FU).

### 4.4. Materials Characterization 

X-Ray diffraction (XRD) patterns were acquired using an X’Pert PRO diffractometer equipped with Cu−Kα radiation (Rigaku Corporation, Tokyo, Japan). Transmission electron microscopy (TEM) images were obtained by using a JEM-2100F transmission electron microscope (Japan Electron Optics Laboratory Co., Ltd., Tokyo, Japan). X-Ray photoelectron spectroscopy (XPS) measurements were performed using a PerkinElmer PHI 5000 ESCT System (PerkinElmer Inc., Massachusetts, Waltham, MA, USA) with a monochromatic Al Kα source with binding energies calibrated against the C 1s peak at 284.8 eV. The specific surface area (S_BET_) was determined by Brunauer–Emmett–Teller (BET) method using a Micrometrics Autosorb-TriStar II Apparatus (Micromeritics Instrument Corporation, Norcross, GA, USA) and obtained from the desorption data. The scanning electron microscopy (SEM) images were observed on a JSM-7001F scanning electron microscope (Japan Electron Optics Laboratory Co., Ltd., Tokyo, Japan). Zeta potential measurements were conducted with a dynamic light scattering analyzer on a Malvern Instruments (Malvern Panalytical Ltd., Malvern, Worcestershire, UK).

### 4.5. Drug Loading and Release Study

The drug loading capacity (DL) of the Fe-MOF material was determined as follows: First, a standard curve for 5-FU was established by preparing gradient concentration solutions of known 5-FU concentrations. The chromatographic peak areas of these solutions were measured using high-performance liquid chromatography (HPLC; detection wavelength: 265 nm). The chromatographic separation was performed using a C18 reversed-phase column (250 × 4.6 mm, 5 μm) with a mobile phase of water-methanol (90:10, *v*/*v*) at a flow rate of 1.0 mL/min. Linear fitting of peak area versus concentration yielded the standard curve equation. During the preparation of Fe-MOF@FU nanomaterials, all supernatant was collected via centrifugation. The peak area of unbound 5-FU in the supernatant was detected under identical HPLC conditions, and the total amount of unbound drug was calculated based on the standard curve. The drug loading capacity was ultimately determined by the difference between the total amount of drug added and the amount of unbound drug, as calculated by Equation (1).

(1) $$\mathrm{DL}\%=\frac{W_{total}-W_{free}}{W_{NS}}$$

where *W_total_* is the total drug mass; *W_free_* represents the free drug mass; *W_NS_* represents the mass of the final Fe-MOF@FU product.

The in vitro drug release performance of the drug-loaded nanomaterials was determined as follows: Precisely weighed Fe-MOF@FU nanomaterials were dispersed in phosphate-buffered saline (PBS) at different pH values (5.0, 6.3, 7.4). The suspensions were transferred into dialysis bags (MWCO: 1000 Da), sealed, and immersed in PBS release media at corresponding pH. The dialysis devices were placed in a thermostatic oscillating water bath at 37 °C with continuous shaking at 100 rpm. At predetermined time intervals, samples were withdrawn (with equal volumes of pre-warmed PBS at identical pH replenished simultaneously). The chromatographic peak areas of 5-FU in the samples were detected by HPLC, and the cumulative drug release was calculated based on the standard curve, as detailed in Equation (2).

(2) $$E=\frac{V_{0}C_{n}+V_{1}\sum_{i=1}^{n-1}C_{i}}{MX}$$

where E represents the cumulative drug release rate; *V*_0_ denotes the total volume of the solution; *V*_1_ is the volume sampled each time; *C_i_* indicates the mass concentration of 5-FU in the solution during the *i*-th sampling (unit: μg/mL); *n* is the number of sampling times; *M* refers to the mass of the drug-loaded material (unit: mg); and *X* represents the drug loading percentage (%).

### 4.6. Fe^2+^ Release in Fe-MOF Materials Study

Based on the pH-responsive iron release mechanism, the Fe^2+^ release from Fe-MOF in PBS at different pH values (5.0, 6.3, 7.4) after 12 h reaction was quantitatively determined using the KMnO_4_ redox titration method: The supernatant obtained by centrifugation was titrated with 0.1 M KMnO_4_ standard solution. Quantitative analysis was performed according to the reaction: 5Fe^2+^ + MnO_4_^−^ + 8H^+^ → 5Fe^3+^ + Mn^2+^ + 4H_2_O, using the transition from colorless to faint purple (endpoint color persisting ≥ 60 s) as the titration endpoint. The Fe^2+^ release amount was calculated based on the above equation to evaluate its potential catalytic activity in generating hydroxyl radicals (·OH) via the Fenton reaction.

### 4.7. Peroxidase-like Activity and Kinetic Experiments

To investigate the ·OH generation catalytic capability of Fe-MOF nanomaterials under different pH conditions and its glutathione (GSH) regulation effect, the following experiments were designed: 400 μg of Fe-MOF was dispersed in 2 mL of PBS buffer (pH = 5.0, 6.3, 7.4) containing H_2_O_2_ (100 mM) and TMB (5 mM). The pH dependence of peroxidase-like (POD) activity was quantitatively characterized by measuring the changes in absorption spectra between 500 and 800 nm after a specific reaction time. Further, using the same reaction system, 2 mM GSH was added for co-reaction. The full-wavelength absorption spectra were measured by the same method to analyze the inhibitory or enhancing effect of GSH on the POD catalytic activity of Fe-MOF.

In kinetic experiments, the Michaelis constant (*K*_m_) is defined as the substrate concentration at half of the maximum reaction rate. *K*_m_ reflects the affinity of the nanozyme for the substrate. The maximum reaction rate (*V*_max_) was observed at saturated substrate concentrations. The initial reaction rate (*V*_0_) and substrate concentration were fitted to the Michaelis-Menten equation to calculate the kinetic constants *K*_m_ and *V*_max_.

(3) $$V_{0}=\frac{V_{max}[S]}{K_{m}+[S]}$$

(4) $$K_{cat}=\frac{V_{max}}{\left[E\right]}$$

where *V*_0_ represents the initial reaction rate, [*S*] denotes the substrate concentration (H_2_O_2_), [*E*] is the molar concentration of Fe-MOF.

### 4.8. Cell Culture

The 4T1 cell line used in experiments were cultured at 37 °C in a humidified incubator with 5% CO_2_. All culture consumables were sterile and single-use. The complete growth medium comprised: 90% RPMI-1640 basal medium, 10% fetal bovine serum (FBS), and 1% penicillin-streptomycin solution.

### 4.9. Cytotoxicity Assessment

The cytotoxicity of Fe-MOF was evaluated using the CCK-8 assay. 4T1 cells were seeded into 96-well plates at an appropriate density and cultured for 12 h to ensure adhesion. The original medium was aspirated and replaced with complete growth medium containing Fe-MOF at varying concentrations (5, 15, 25, 50, 100, 200 μg/mL). After 12 h or 24 h incubation, cell viability was measured to assess material toxicity and determine subsequent therapeutic concentrations. For in vitro therapeutic evaluation, 4T1 cells were cultured identically. Experimental groups included: (1) CDT: 100 μM H_2_O_2_ + Fe-MOF; (2) 5-FU only; (3) 100 μM H_2_O_2_ + 5-FU; (4) Fe-MOF@FU; (5) Combined therapy: 100 μM H_2_O_2_ + Fe-MOF@FU. Concentrations of 5-FU and Fe-MOF@FU were consistent with material toxicity assessment. Post-adhesion, treatments were administered for 12 h or 24 h. After treatment, 10% (*v*/*v*) CCK-8 reagent was added to each well and incubated for 1.5 h protected from light. Optical density (OD) at 450 nm was measured using a microplate reader (Tecan, Beijing, China), and cell viability was calculated (Equation (5)).

(5) $$\mathrm{Cell}\;\mathrm{viability}\;(\%)=\frac{{(OD}_{experimental\;group\;}-{OD}_{control\;group})}{\left({OD}_{blank\;group\;}-{\;OD}_{control\;group}\right)}\times 100\%$$

where *OD*_experimental group_: absorbance of experimental hole (including cell, medium, CCK-8 solution and drug solution); *OD*_control group_: absorbance of negative control group (including cells, culture medium, CCK-8 solution, no drugs); *OD*_blank group_: absorbance of blank group (including culture medium and CCK-8 solution, without cells and drugs).

### 4.10. Cellular Uptake of Materials

To quantitatively characterize the uptake kinetics of Fe-MOF nanomaterials by 4T1 cells, a time-gradient uptake assay was designed based on the chromogenic principle wherein potassium hexacyanoferrate (II) (K_3_[Fe(CN)_6_]) reacts with intracellular iron ions to form insoluble Prussian blue precipitates (KFe_2_[CN]_6_). The procedure was as follows: 4T1 cell suspensions were seeded in 12-well plates at 500 μL per well. After adhesion, the supernatant was aspirated and replaced with 500 μL of medium containing 200 μg/mL Fe-MOF, followed by incubation for 0, 2, 4, 8, 12, or 24 h. At each time point, uptake was terminated by removing the material-containing medium. Cells were washed 1–2 times with PBS to remove extracellular residues. Subsequently, 500 μL of K_3_[Fe(CN)_6_] solution (2.0 mM in PBS) was added to each well and incubated at 37 °C for 1 h. Formation of intracellular blue precipitates was observed and imaged using an inverted fluorescence microscope.

### 4.11. Cell Migration Assay

To evaluate the concentration-dependent inhibitory effect of Fe-MOF@FU on 4T1 cell migration, a scratch wound healing model was employed. 4T1 cells were seeded in 6-well plates and cultured until >90% confluency. Sterile 200 μL pipette tips were used to create uniform scratches perpendicular to the plate surface. After three gentle PBS washes to remove detached cells, initial scratch images were captured using an inverted microscope. The medium was then replaced with low-serum medium containing 2% FBS. Experimental groups received gradient concentrations of Fe-MOF@FU (5, 25, 100, 200 μg/mL), while the control group received equal volumes of fresh medium. All groups were incubated at 37 °C under 5% CO_2_. At 4, 12, and 24 h post-treatment, medium was aspirated, cells were PBS-washed, and images were acquired at identical scratch locations.

### 4.12. Calcein-AM/Propidium Iodide (PI) Staining

Cell viability was assessed using the Calcein-AM/PI double staining method. The specific procedure was as follows: 4T1 cells were seeded into 20 mm confocal laser dishes and incubated for 12 h to allow for full cell adhesion. The original culture medium was then discarded, and the cells were washed three times with PBS buffer. The following treatment groups were established: (1) Control group: RPMI-1640 basal medium; (2) H_2_O_2_ control group: 100 μM H_2_O_2_; (3) Material-only group: 200 μg/mL Fe-MOF; (4) Drug-only group: 116 μg/mL 5-FU; (5) Drug-loaded material group: 200 μg/mL Fe-MOF@FU; (6) Drug treatment group: 100 μM H_2_O_2_ + 116 μg/mL 5-FU; (7) CDT group: 100 μM H_2_O_2_ + 200 μg/mL Fe-MOF; (8) Combination therapy group: 100 μM H_2_O_2_ + 200 μg/mL Fe-MOF@FU. After the respective interventions for each group were completed, the cells were washed three times with PBS buffer. Subsequently, under light-protected conditions, the cells were co-incubated with Calcein-AM/PI staining solution for 20 min. After removing the staining solution, the cells were washed again with PBS three times. Finally, fluorescent images were captured using a confocal laser scanning microscope (Leica, Wetzlar, Germany), and quantitative analysis was performed using Image software (LAS_X_4.7.0).

### 4.13. Intracellular Reactive Oxygen Species (ROS) Detection

Following cell culture and group interventions (group settings identical to the Calcein-AM/PI double staining experiment), the culture medium from each treatment group was removed, and the cells were washed three times with ice-cold PBS buffer to eliminate residual components. Subsequently, the ROS-specific fluorescent probe DCFH-DA was added, and the cells were incubated at 37 °C in a light-protected environment for 30 min to allow sufficient probe penetration into live cells. After incubation, the staining solution was discarded, and the cells were washed three times with PBS buffer to thoroughly remove any uninternalized free probe. Finally, intracellular green fluorescent images were captured using a confocal laser scanning microscope, and the fluorescence intensity was quantitatively analyzed using ImageJ software (LAS_X_4.7.0).

### 4.14. Flow Cytometric Apoptosis Assay

Cell apoptosis rates were quantitatively analyzed using Annexin V-FITC/PI double staining combined with flow cytometry. 4T1 cells were seeded into 6-well plates, with experimental grouping and treatment conditions consistent with the Calcein-AM/PI staining method. When cells reached approximately 80% confluence, the medium containing treatment materials was discarded, and cells were washed once with ice-cold PBS buffer. Cells were then digested with 0.25% trypsin (without EDTA) at 37 °C for 3 min, followed by termination of digestion using serum-containing medium. The cell suspension was collected, centrifuged, and resuspended in PBS. After two additional washing cycles via centrifugation, the cell pellet was resuspended in ice-cold 1× Binding Buffer. According to the manufacturer’s instructions, Annexin V-FITC and PI were sequentially added followed by incubation in the dark. Finally, the proportion of apoptotic cells was detected using a flow cytometer, with all experiments independently repeated three times.

### 4.15. Biocompatibility Evaluation

#### 4.15.1. Hemolysis Assay

Hemolysis testing was performed using blood collected from healthy BALB/c mice via retro-orbital bleeding into anticoagulant-containing tubes. The blood was centrifuged at 5000 r/min for 5 min, and the supernatant was discarded. Erythrocytes were repeatedly washed under identical centrifugation conditions until the supernatant became clear and colorless. The pelleted erythrocytes were collected and resuspended in PBS buffer to prepare a 2% (*v*/*v*) erythrocyte suspension. Eight experimental groups were established: positive control (ultrapure water), negative control (PBS solution), and PBS-diluted gradient concentrations of Fe-MOF and Fe-MOF@FU (5, 15, 25, 50, 100, 200 μg/mL). For each group, 0.8 mL of the sample solution was mixed with 0.2 mL of the erythrocyte suspension to form a 1.0 mL homogeneous mixture. The mixtures were incubated at 37 °C in a constant-temperature shaking water bath for 2 h. After incubation, the samples were centrifuged at 5000 r/min for 5 min. The supernatant was transferred to a 96-well plate, and the absorbance (OD value) at 570 nm was measured using a microplate reader. The Hemolysis Percentage (HP) was calculated according to Formula (6).

(6) $$\mathrm{HP}\;(\%)=\;\frac{{(OD}_{sample}-{OD}_{negative})}{{(OD}_{positive}-{OD}_{negative})}\times 100\%$$

#### 4.15.2. Mouse Organ Sectioning and Liver/Kidney Function Index Measurement

All animal procedures were approved by the Ethics Committee of Inner Mongolia Medical University (Approval No. YKD202501237). Eight healthy female BALB/c mice were selected and randomly divided into a drug-loaded material group (*n* = 4) and a blank control group (*n* = 4). The drug-loaded material group received an intraperitoneal injection of 0.35 mL of Fe-MOF@5-FU suspension (200 μg/mL, concentration consistent with the treatment group), while the blank control group was injected with an equal volume of PBS buffer. After 48 h of administration, one mouse from each group was randomly euthanized. Organs (heart, liver, spleen, lung, and kidney) were harvested, fixed in 4% paraformaldehyde, and processed into paraffin sections. Hematoxylin and eosin (H&E) staining was performed to compare histopathological differences between the two groups. Subsequently, one additional mouse was randomly selected from each group (including one mouse from the remaining animals per group). Blood was collected via retro-orbital bleeding into clot activator tubes. Serum was separated by centrifugation at 3000 r/min for 5 min and used to measure liver and kidney function indices, including alanine aminotransferase (ALT), aspartate aminotransferase (AST), alkaline phosphatase (ALP), urea (UREA), and creatinine (CREA).

## Data Availability

All data and materials are available on request from the corresponding authors. The data are not publicly available due to ongoing research using a part of the data.

## Supplementary Materials

The following supporting information can be downloaded at: https://www.mdpi.com/article/10.3390/gels11090750/s1, Figure S1. Hydrodynamic size of Fe-MOF after dispersing into DI PBS and RPMI-1640 (with 10% FBS) for five days. Figure S2. Zeta potentials of Fe-MOF and Fe-MOF@FU in deionized water. Figure S3. Full-survey XPS spectrum of Fe-MOF. Figure S4. Standard curve of 5-FU. Table S1. Comparison of specific surface area and drug loading capacity between Fe/Zn-bimetallic MOF (this work) and other representative MOF supports. Table S2. Original data on the Michaelis-Menten kinetics of Fe-MOF. Table S3. List of abbreviations. References [52,53,54,55,56,57] are cited in the supplementary materials.
