# Supplementary material for: Concave-Octahedral Fe2+-Rich Fe-MOF/FU Nano-Blocks with Enhanced pH-Responsive Nanozyme Activity Toward Stimuli-Responsive Gels for Chemo-Chemodynamic Synergistic Therapy
Source: Gels. 2025 Sep 17;11(9):750. doi: 10.3390/gels11090750 (PMC12469758; doi:10.3390/gels11090750)
Supplement: Supplementary file 1 [file gels-11-00750-s001.zip › gels-3851204-supplementary.pdf]

Supporting Information

Concave-Octahedral Fe^{2+}-Rich Fe-MOF/FU Nano-Blocks with Enhanced pH-Responsive Nanozyme Activity Toward Stimuli-Responsive Gels for Chemo-Chemodynamic Synergistic Therapy

Desheng Wang [1,†], Changjin Xu [1,*,†], Laibing Wang [1], Herima Qi [1], Riqing Cheng [1], Liang Bao [2], Huiqing Guo [1,*] and Shikui Wu [1,*]

[1] College of Pharmacy, Inner Mongolia Medical University, Hohhot 010110, China
[2] Medical Innovation Center for Nationalities, Inner Mongolia Medical University, Hohhot 010110, China
* Correspondence: changjin.xu@immu.edu.cn (C.X.); ghq5@163.com (H.G.); wushikui@immu.edu.cn (S.W.)
† These authors contributed equally to this work.

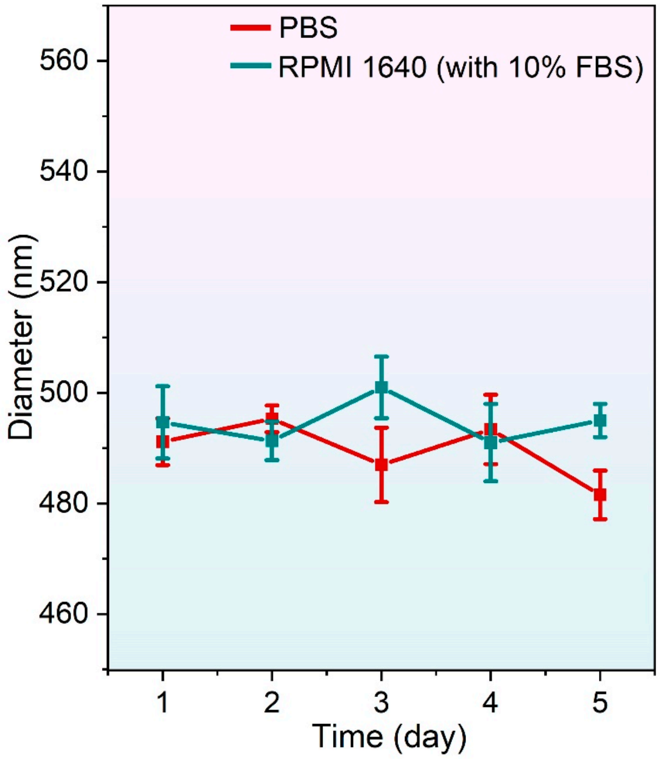

Figure S1. Hydrodynamic size of Fe-MOF after dispersing into DI PBS and RPMI-1640 (with 10% FBS) for five days.

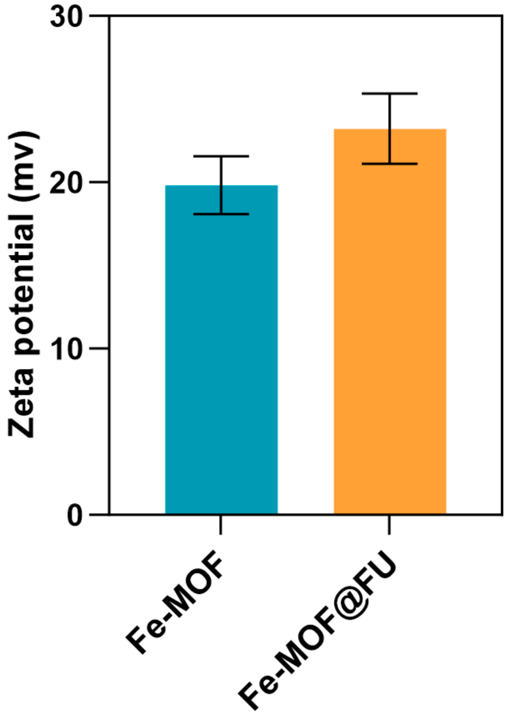

Figure S2. Zeta potentials of Fe-MOF and Fe-MOF@FU in deionized water.

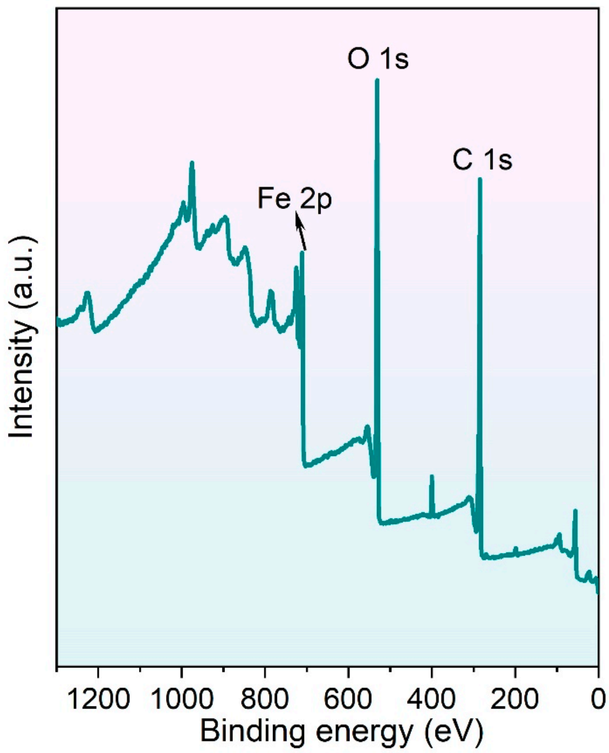

Figure S3. Full-survey XPS spectrum of Fe-MOF.

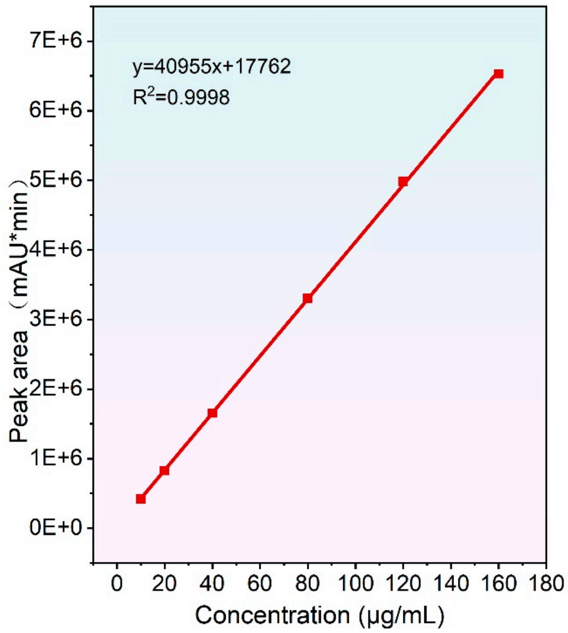

Figure S4. Standard curve of 5-FU.

Table S1. Comparison of specific surface area and drug loading capacity between Fe/Zn-bimetallic MOF (this work) and other representative MOF supports.

Materials	S_{BET} m^2/g	Drug loading	References
Fe-MOF	559	58.7%	In this work
Fe_3O_4@CS@UIO-66-NH_2(Zr)	91.182	14%	[1]
AuNPs@ZIF-8/ZIF-67	218	40%	[2]
MOF-NCs	78.3428	55.8%	[3]
FeTCPP/Fe_2O_3-MOF	154.3	18.7%	[4]
MSNCs	249.2	23.6±1.2%	[5]
L/HKUST-1	98.85	49.8%	[6]

Table S2. Original data on the Michaelis-Menten kinetics of Fe-MOF.

H_2O_2 concentration (μM)	Absorbance (△A)		
5	0.0091	0.0087	0.0093
10	0.0192	0.02	0.0212
20	0.0257	0.0236	0.02359
40	0.0383	0.0387	0.0392
80	0.0401	0.0396	0.0412
100	0.041	0.053	0.042

Table S3. List of abbreviations.

Full name of abbreviation	Abbreviation	Full name of abbreviation	Abbreviation
Fe-based metal−organic framework	Fe-MOF	5-Fluorouracil	5-FU
Glutathione	GSH	Tumor microenvironment	TME
Chemodynamic Therapy	CDT	Ferric chloride hexahydrate	$FeCl_3 \cdot 6H_2O$
Zinc nitrate hexahydrate	$Zn(NO_3)_2 \cdot 6H_2O$	*N*,*N*-dimethylformamide	DMF
Potassium ferrocyanide	$K_4Fe(CN)_6$	Terephthalic acid	PTA
Polyvinylpyrrolidone	PVP	Fetal bovine serum	FBS
Calcein acetoxymethylester	Calcein-AM	Propidium iodide	PI
2′,7′-dichlorodihydrofluorescein diacetate	DCFH-DA	3,3′,5,5′-tetramethylbenzidine	TMB

References

1. Yaghoubian, A.; Setoodehkhah, M.; Parsa, F. Investigation of Pantoprazole Loading and Release from a Magnetic-Coated Chitosan-Modified Zirconium-Based Metal–Organic Framework (MOF) as a Nanocarrier in Targeted Drug Delivery Systems. *RSC Adv.* **2024**, *14*, 26091–26102.
2. Rananaware, P.; Pandit, P.; Brahmkhatri, V. Gold Nanoparticle Encapsulated Hybrid MOF: Synthesis, Characterization, and Co-Drug Delivery of 5-Fluorouracil and Curcumin. *Discov. Nano* **2024**, *19*, 201.
3. Moaness, M.; Mabrouk, M.; Ahmed, M.M.; Das, D.B.; Beherei, H.H. Novel Zinc-Silver Nanocages for Drug Delivery and Wound Healing: Preparation, Characterization and Antimicrobial Activities. *Int. J. Pharm.* **2022**, *616*, 121559.
4. Zhao, Y.; Wang, J.; Cai, X.; Ding, P.; Lv, H.; Pei, R. Metal–Organic Frameworks with Enhanced Photodynamic Therapy: Synthesis, Erythrocyte Membrane Camouflage, and Aptamer-Targeted Aggregation. *ACS Appl. Mater. Interfaces* **2020**, *12*, 23697–23706.
5. Wang, S.; Liu, X.; Chen, S.; Liu, Z.; Zhang, X.; Liang, X.-J.; Li, L. Regulation of Ca^{2+} Signaling for Drug-Resistant Breast Cancer Therapy with Mesoporous Silica Nanocapsule Encapsulated Doxorubicin/SiRNA Cocktail. *ACS Nano* **2019**, *13*, 274–283.
6. Djahaniani, H.; Ghavidel, N.; Kazemian, H. Green and Facile Synthesis of Lignin/HKUST-1 as a Novel Hybrid Biopolymer Metal-Organic-Framework for a PH-Controlled Drug Release System. *Int. J. Biol. Macromol.* **2023**, *242*, 124627.
